# HDA-2-Containing Complex Is Required for Activation of *Catalase-3* Expression in Neurospora crassa

**DOI:** 10.1128/mbio.01351-22

**Published:** 2022-06-14

**Authors:** Lingaonan He, Zeyu Duan, Muqun Yu, Shaohua Qi, Ying Wang, Huiqiang Lou, Qun He

**Affiliations:** a State Key Laboratory of Agro-biotechnology, College of Biological Sciences, China Agricultural University, Beijing, China; b MOA Key Laboratory of Soil Microbiology, College of Biological Sciences, China Agricultural University, Beijing, China; University of Georgia

**Keywords:** HDA-2, Histone H4 acetylation, Catalase-3, *Neurospora crassa*

## Abstract

It is essential for aerobic organisms to maintain the homeostasis of intracellular reactive oxygen species (ROS) for survival and adaptation to the environment. In line with other eukaryotes, the catalase of Neurospora crassa is an important enzyme for clearing ROS, and its expression is tightly regulated by the growth phase and various oxidative stresses. Our study reveals that, in N. crassa, histone deacetylase 2 (HDA-2) and its catalytic activity positively regulate the expression of the *catalase-3* (*cat-3*) gene. HDA-2, SIF-2, and SNT-1 may form a subcomplex with such a regulation role. As expected, deletion of HDA-2 or SIF-2 subunit increased acetylation levels of histone H4, indicating that loss of HDA-2 complex fails to deacetylate H4 at the *cat-3* locus. Furthermore, loss of HDA-2 or its catalytic activity led to dramatic decreases of TFIIB and RNA polymerase II (RNAP II) recruitment at the *cat-3* locus and also resulted in high deposition of H2A.Z at the promoter and transcription start site (TSS) regions of the *cat-3* gene. Collectively, this study strongly demonstrates that the HDA-2-containing complex activates the transcription of the *cat-3* gene by facilitating preinitiation complex (PIC) assembly and antagonizing the inhibition of H2A.Z at the *cat-3* locus through H4 acetylation.

## INTRODUCTION

Aerobic organisms produce reactive oxygen species (ROS) as by-products through the electron transport chain during intracellular and extracellular redox reactions. ROS mainly consist of singlet oxygen, superoxide radical, hydrogen peroxide, and hydroxyl radicals. Although ROS have pivotal roles in cell signal transduction and homeostasis (1–4), they also pose a serious threat to cells if the intracellular ROS levels deviate from the optimal amount (5–8). Excessive ROS can cause extensive oxidation damage to proteins, unsaturated fatty acids, and DNA, resulting in dysfunction or death of cells (5, 6, 8, 9). To counteract the damaging effects of ROS, eukaryotic cells have evolved various antioxidation strategies which convert harmful ROS into harmless constituents to cells. Superoxide dismutase (SOD), catalases (CAT), and peroxidases are three main types of enzymes involved in ROS clearance, among which SOD and CAT are the primary antioxidants in nearly all living cells exposed to oxygen (2, 3, 10, 11). These clearance mechanisms are highly conserved from bacteria to mammals (12).

The filamentous fungus Neurospora crassa possesses four catalase genes, *cat-1*, *cat-2*, *cat-3*, and *cct-1/cat-4* (13–16). Among them, *cat-1*, *cat-2*, and *cat-3* are inducible by oxidative stress and differentially expressed during the asexual life cycle (16, 17). Previous study showed that CAT-3 is the key catalase in growing hyphae whose function could not be replaced by other catalases (18). Furthermore, CAT-3 protein can be induced by various oxidative stresses (19). For example, H_2_O_2_ treatment can stimulate *cat-3* expression by elevating histone acetylation levels of the *cat-3* locus (20). Consistent with this, CPC1/GCN4 and the histone acetyltransferase GCN5 have been proved to positively regulate the expression of *cat-3* (21). Furthermore, our previous research showed that histone variant H2A.Z is also a critical regulator for *cat-3* transcription. SWR complex-mediated H2A.Z deposition and INO80 complex-mediated H2A.Z removal both significantly influence *cat-3* transcription (22, 23). These results suggest that the chromatin structure and histone modifications play major roles in regulating the inducible expression of the *cat-3* gene.

The dynamics of histone acetylation are controlled by the antagonistic roles of histone acetyltransferases (HATs) and histone deacetylases (HDACs). In general, HDACs frequently serve as corepressors to inactivate transcription. Hos2 has been identified as a HDAC subunit in the Set3 complex, accompanied by Set3, Sif2, Snt1, YIL112w, Cpr1, and another histone deacetylase, Hst1 (24). Unlike the classical repressive role of most HDACs, Saccharomyces cerevisiae Hos2 can bind to and deacetylate histone in the coding region of active genes (25). Genetic evidence has shown that Set3 and the deacetylase activity of Hos2 promote the efficient activation of the *GAL1* gene by deacetylating specific lysines in histones H3 and H4 (25, 26). Further study showed that the Set3 complex deacetylates histone at the 5′ ends of actively transcribed genes (27). In mammalian cells, the Set3 complex was proposed to be a functional homolog of the NCoR/SMRT complex, which forms a stable complex with histone deacetylase HDAC3 and TBL1. The deacetylases HDAC3 and TBL1 are mammalian homologs of Hos2 and Sif2, which are core subunits of the S. cerevisiae Set3 complex (28, 29). In addition, the Snt1 subunit of the Set3 complex shares the SANT domain with NCoR/SMRT (24, 30). Even though HDAC3 can act as a transcriptional corepressor, it is required for transcriptional activation in a class of retinoic acid response elements (31, 32). These results strongly suggest that the conserved enzymatic activity of the Set3 complex and NCoR/SMRT complex appear to be the Hos2 and HDAC3 subunits, which deacetylate histone H3 and H4 at the actively transcribed genes.

In budding yeast, Htz1/H2A.Z preferentially occupies the promoter regions of two transcriptionally inactive but inducible genes, *GAL1* and *PHO5* (33–37). Further study revealed that *htz1Δ* is synthetic sick with each core subunit of the Set3 complex, such as SET3, HOS2, SIF2, and SNT1, suggesting overlapping roles of them in some biological processes. Interestingly, the severe slow growth phenotype of *htz1Δ set3Δ* was partially suppressed by further deletion of the chromatin remodeler *SWR1* gene (38). These results suggested that Set3/Hos2 histone deacetylase has previously unrecognized functions in the dynamic deposition or remodeling of nucleosomes containing H2A.Z. A recent study identified HDA-2 as a positive regulator for *cat-3* transcription in Trichoderma atroviride (39). However, it is not clear whether HDA-2 is involved in regulation of *cat-3* gene expression in N. crassa.

Here, we revealed that in N. crassa, histone deacetylase 2 (HDA-2) positively regulates *cat-3* gene expression, and such a regulatory role depends on its catalytic activity. Immunoprecipitation data confirmed the interaction among N. crassa HDA-2, SIF-2, and SNT-1 proteins. Like HDA-2, deletion of the SIF-2 or SNT-1 subunit in HDA-2-containing complex downregulates *cat-3* expression. The acetylation levels of histone H4 were increased in these mutants, indicating that loss of the HDA-2 complex fails to deacetylate H4 at the *cat-3* locus. Chromatin immunoprecipitation (ChIP) assays revealed that loss of HDA-2 or its catalytic activity results in defective assembly of the preinitiation complex (PIC) and high deposition of H2A.Z at the *cat-3* locus. Taken together, our results demonstrate that the HDA-2 complex is critical for *cat-3* activation.

## RESULTS

### HDA-2, SIF-2, and SNT-1 may form a subcomplex for resisting H_2_O_2_-induced ROS stress.

**FIG 1:**
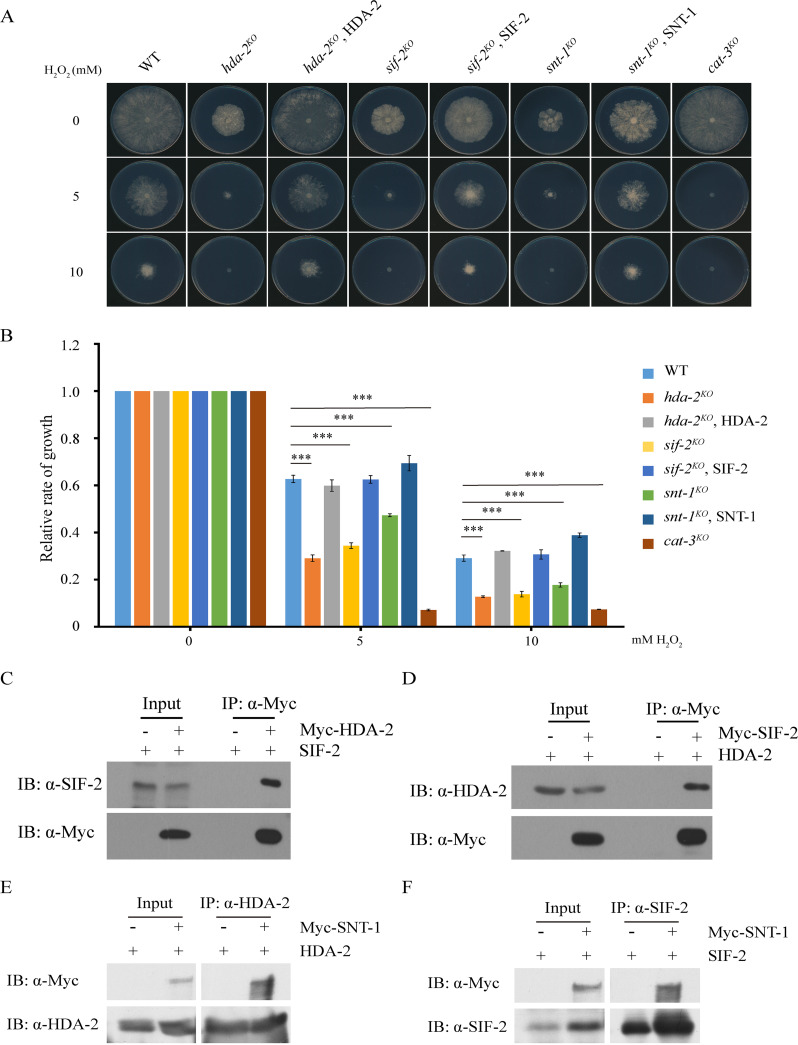
HDA-2, SIF-2, and SNT-1 may form a subcomplex for resisting H_2_O_2_-induced ROS stress. (A) Mycelial growth of the wild type (WT) and *cat-3^KO^*, *hda-2^KO^*, *sif-2^KO^*, or *snt-1^KO^* and HDA-2, SIF-2, or SNT-1 transformants in plates with 0, 5, or 10 mM H_2_O_2_ as indicated. Cultures were inoculated in plates at 25°C under constant light. (B) Quantitative results for relative growth rate of the WT, *cat-3^KO^*, *hda-2^KO^*, *sif-2^KO^*, or *snt-1^KO^* and HDA-2, SIF-2, or SNT-1 transformants. Error bars show standard deviations (SD; *n *= 3). Significance was assessed by using a two-tailed *t* test. ***, *P* < 0.001 versus WT. (C and D) Mapping of endogenous SIF-2 or HDA-2 responsible for the interaction with ectopically expressing wild-type Myc-HDA-2 in *hda-2^KO^* strain or Myc-SIF-2 in *sif-2^KO^* strain, respectively. In immunoprecipitation assays with anti-Myc antibody (α-Myc), the eluates were detected by Western blotting using anti-SIF-2 (α-SIF-2) and anti-Myc (α-Myc) antibodies (C) or anti-HDA-2 (α-HDA-2) and anti-Myc (α-Myc) antibodies (D), respectively. (E and F) Immunoprecipitation assays showing the interaction between Myc-SNT-1 and endogenous HDA-2 (E) or SIF-2 (F) protein, respectively.

To identify the factors that contribute to resisting H_2_O_2_-induced ROS stress, we performed H_2_O_2_ sensitivity assays to screen N. crassa knockout mutants. We found that a strain with deletion of the *hda-2* gene (NCU02795) exhibited a severe H_2_O_2_ sensitivity phenotype compared to that of the wild-type (WT) strain (Fig. 1A and B). HDA-2 is a class I histone deacetylase and is the homolog of Saccharomyces cerevisiae Hos2 and Schizosaccharomyces pombe Phd1. In S. cerevisiae, Hos2p is the deacetylase subunit of the Set3 complex (24). Therefore, we searched N. crassa homologs of the other subunits in the S. cerevisiae Set3 complex through sequence alignment and identified SET-4 (SET3), NST-1 (HST1), NCU10346 (SNT1), NCU00388 (YIL112w), NCU06838 (SIF2), and NCU01200 (CPR1) (see Fig. S1A in the supplemental material).

10.1128/mbio.01351-22.1 FIG S1 **Subunits of the Set3 complex in** **Saccharomyces cerevisiae** **and the interaction of their homologous proteins in** **Neurospora crassa.** (A) Search for homologs of the S. cerevisiae Set3 complex in N. crassa by sequence alignment yielded information on seven proteins and their conserved domains. (B to I) Mapping of endogenous HDA-2 or SIF-2 responsible for the interaction with ectopically expressing Myc-SET-4, Myc-NST-1, Myc-YIL112w, or Myc-CPR-1 in the WT strain. For immunoprecipitation assays with anti-HDA-2 antibody (α-HDA-2) or anti-SIF-2 antibody (α-SIF-2), the eluates were detected by Western blotting using α-HDA-2 and α-Myc antibodies (B, D, F, and H) or α-SIF-2 and α-Myc antibodies (C, E, G, and I), respectively. Download FIG S1, TIF file, 1.1 MB. Copyright © 2022 He et al. 2022 He et al. https://creativecommons.org/licenses/by/4.0/ This content is distributed under the terms of the Creative Commons Attribution 4.0 International license.

To systematically analyze the function of each subunit in H_2_O_2_-induced ROS stress, we tried to generate deletion mutants of these genes by gene replacement in the *ku70^RIP^* strain. However, we could not obtain the homokaryotic deletion strains of the Yil112w (NCU00388) or Cpr1 (NCU01200) gene, suggesting that these two genes are essential to cell viability in N. crassa. As shown in Fig. 1A and B, deletion of *sif-2* (NCU06838) or *snt-1* (NCU10346) resulted in a similar H_2_O_2_ sensitivity phenotype with the *hda-2^KO^* strain, indicating that these subunits probably cooperate to resist H_2_O_2_-induced ROS stress. To further confirm the H_2_O_2_ sensitivity phenotype of these knockout strains, we generated the complementary strains by transforming Myc-tagged constructs of subunit proteins into each corresponding knockout strain. As expected, ectopic expression of Myc-tagged proteins restored the H_2_O_2_ sensitive phenotypes of *hda-2^KO^*, *sif-2^KO^*, or *snt-1^KO^* strains to those of WT strains (Fig. 1A and B), indicating that the H_2_O_2_ sensitivity phenotype of each mutant was due to the deletion of each subunit gene. However, the strains with deletion of *nst-1* (NCU04737) or *set-4* (NCU04389) exhibited similar H_2_O_2_ sensitivity as the WT strain (see Fig. S2A in the supplemental material), suggesting that NST-1 and SET-4 are not key regulators for resisting H_2_O_2_-induced ROS stress. Therefore, the HDA-2, SIF-2, and SNT-1 proteins play significant roles in responding to H_2_O_2_-induced ROS stress in N. crassa.

10.1128/mbio.01351-22.2 FIG S2 **NST-1 and SET-4 are not key regulators for resisting H_2_O_2_-induced ROS stress.** (A) Growth of the WT, *nst-1^KO^*, and *set-4^KO^* in plates under the treatment of 0 or 10 mM H_2_O_2_. (B) Western blot showing the levels of CAT-3 protein in the WT, *nst-1^KO^*, and *set-4^KO^*. The membrane stained by Coomassie blue represents the total protein in each sample and acted as loading control for Western blotting. (C) Catalase activity assay. Crude extracts from the WT, *nst-1^KO^*, and *set-4^KO^* were subjected to native-PAGE, and the catalase activities were determined with the in-gel assay. (D) RT-qPCR analysis showing the levels of *cat-3* mRNA in the WT, *nst-1^KO^*, and *set-4^KO^*. Error bars show SD (*n *= 3). Download FIG S2, TIF file, 0.8 MB. Copyright © 2022 He et al. 2022 He et al. https://creativecommons.org/licenses/by/4.0/ This content is distributed under the terms of the Creative Commons Attribution 4.0 International license.

To confirm the integrity of N. crassa HDA-2-containing complex, we generated HDA-2- and SIF-2-specific antibodies (see Fig. S3A and B in the supplemental material) and examined the interaction between HDA-2 and SIF-2 in *hda-2^KO^*, Myc-HDA-2 and *sif-2^KO^*, Myc-SIF-2 transformants. Immunoprecipitation assays revealed that the Myc-HDA-2 or Myc-SIF-2 protein strongly interacts with endogenous SIF-2 or HDA-2, respectively (Fig. 1C and D). The *snt-1^KO^* strain exhibited similar H_2_O_2_ sensitivity as *hda-2^KO^* and *sif-2^KO^* strains, so we performed immunoprecipitation assays, which revealed that the Myc-SNT-1 protein strongly interacts with endogenous HDA-2 or SIF-2, respectively (Fig. 1E and F). To further confirm whether other homologous proteins of the S. cerevisiae Set3 complex also form complexes in N. crassa, we performed immunoprecipitation assays and found that Myc-SET-4, Myc-NST-1, Myc-YIL112w, and Myc-CPR-1 proteins interact with endogenous HDA-2 or SIF-2 (see Fig. S1B to I in the supplemental material). These results demonstrated that HDA-2, SIF-2, and SNT-1 may form a subcomplex to resist H_2_O_2_-induced oxidative stress in N. crassa.

10.1128/mbio.01351-22.3 FIG S3 **Detection of HDA-2- and SIF-2-specific antibodies.** (A and B) Immunodetection of HDA-2 or SIF-2 protein in WT, *hda-2^KO^* (A), and *sif-2^KO^* or *sif-2^KO^*, SIF-2 (B) strains using the corresponding polyclonal antibody that specifically recognized the HDA-2 and SIF-2 or Myc-SIF-2 proteins in the WT or *sif-2^KO^*, SIF-2 strain, respectively. Download FIG S3, TIF file, 0.4 MB. Copyright © 2022 He et al. 2022 He et al. https://creativecommons.org/licenses/by/4.0/ This content is distributed under the terms of the Creative Commons Attribution 4.0 International license.

### HDA-2-containing complex plays a key role in activation of *cat-3* transcription.

**FIG 2:**
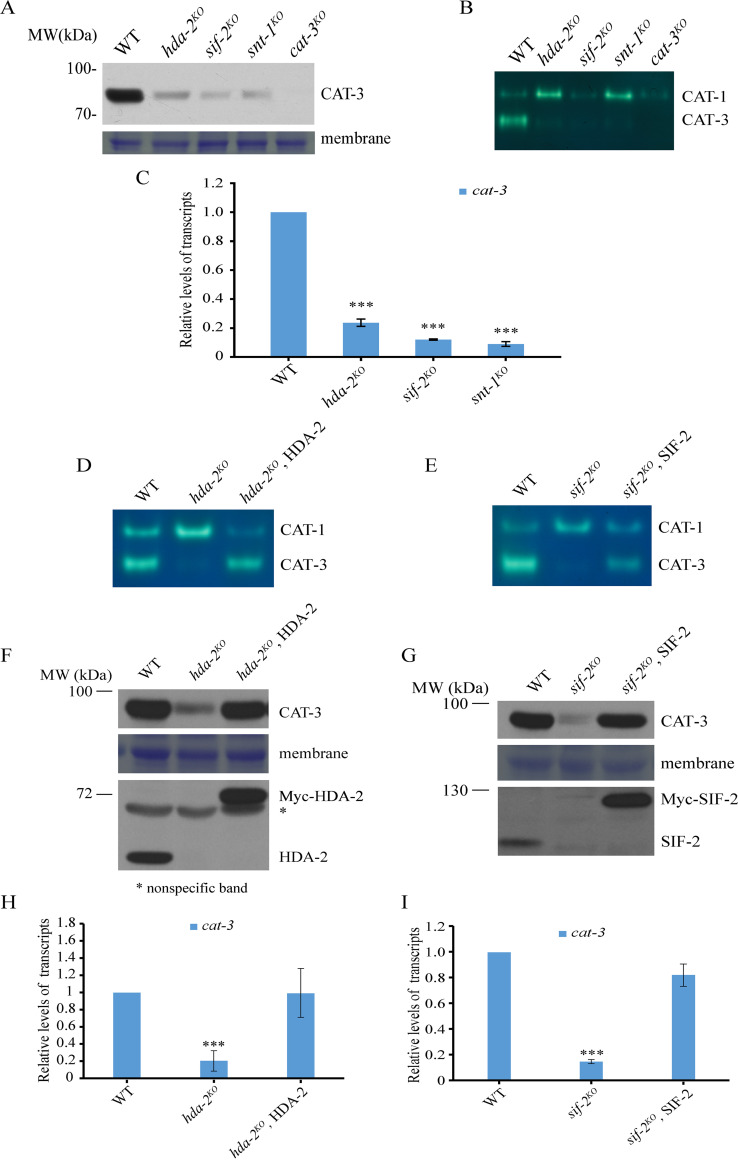
HDA-2-containing complex plays a key role for the activation of *cat-3* transcription. (A) Western blot showing the levels of CAT-3 protein in the WT, *hda-2^KO^*, *sif-2^KO^*, *snt-1^KO^*, and *cat-3^KO^* strains. The membrane stained with Coomassie blue represents the total protein in each sample and acted as a loading control for Western blotting. (B) Catalase activity in-gel assay. Crude extracts from the WT, *hda-2^KO^*, *sif-2^KO^*, *snt-1^KO^*, and *cat-3^KO^* strains were subjected to native-PAGE, and the catalase activity was determined in the in-gel assay. (C) RT-qPCR analysis showing the levels of *cat-3* mRNA in the WT, *hda-2^KO^*, *sif-2^KO^*, and *snt-1^KO^* strains. Error bars show SD (*n *= 3). Significance was assessed by using a two-tailed *t* test. ***, *P* < 0.001 versus WT. (D and E) Catalase activity in-gel assays. Crude extracts from the WT, *hda-2^KO^*, or *sif-2^KO^* and HDA-2 or SIF-2 transformants were subjected to native-PAGE, and the catalase activity was determined with the in-gel assay. (F and G) Western blot showing the levels of CAT-3, HDA-2, or SIF-2 and Myc-HDA-2 or Myc-SIF-2 proteins in the WT, *hda-2^KO^*, or *sif-2^KO^* and HDA-2 or SIF-2 transformants. The membranes stained with Coomassie blue represent the total protein in each sample and acted as a loading control for Western blotting. (H and I) RT-qPCR analyses showing the levels of *cat-3* mRNA in the WT, *hda-2^KO^*, or *sif-2^KO^* and HDA-2 or SIF-2 transformants. Error bars show SD (*n *= 3). Significance was assessed by using a two-tailed *t* test. ***, *P* < 0.001 versus WT.

Since CAT-3 is the major catalase in mycelia, the genetic analysis results above suggest that the HDA-2-containing complex may participate in the regulation of *cat-3* expression in N. crassa. To test this possibility, we performed a Western blot assay to analyze the protein levels of CAT-3 in the WT and each mutant strain. As shown in Fig. 2A (see also Fig. S2B in the supplemental material), the protein levels of CAT-3 in *hda-2^KO^*, *sif-2^KO^*, and *snt-1^KO^* strains were extremely lower than in the WT strain, whereas the protein levels of CAT-3 in the *nst-1^KO^* and *set-4^KO^* strains were similar to that in the WT strain. As expected, an in-gel assay showed that the stained bands corresponding to CAT-3 activity were weaker in *hda-2^KO^*, *sif-2^KO^*, and *snt-1^KO^* mutants than in the WT, *nst-1^KO^*, and *set-4^KO^* strains (Fig. 2B; see also Fig. S2C). Consistent with the protein and catalase activity results, the levels of *cat-3* mRNA in *hda-2^KO^*, *sif-2^KO^*, and *snt-1^KO^* mutants were also much lower than those in the WT, *nst-1^KO^*, and *set-4^KO^* strains (Fig. 2C; see also Fig. S2D). These results suggest that the decreased expression of *cat-3* in these mutants is responsible for their sensitivity to H_2_O_2_-induced ROS stress. In addition, ectopic expression of Myc-tagged HDA-2 or SIF-2 in each corresponding deletion strain restored the levels of CAT-3 activity (Fig. 2D and E) and *cat-3* expression to WT levels (Fig. 2F to I). Taken together, these results demonstrated that HDA-2-containing complex is critical for transcriptional activation of the *cat-3* gene.

### The deacetylase activity of HDA-2 is required for activation of *cat-3* transcription.

**FIG 3:**
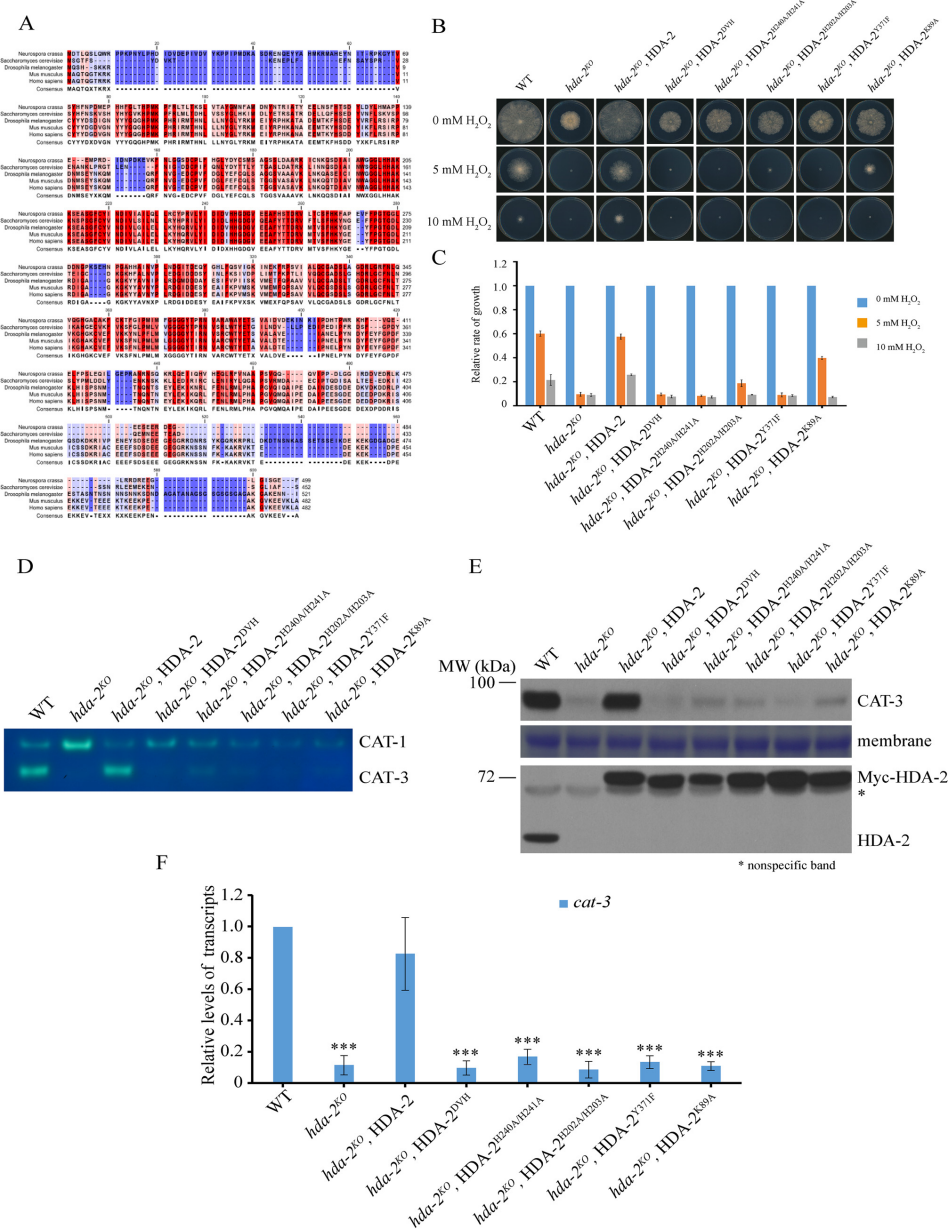
The deacetylase activity of HDA-2 is required for activation of *cat-3* transcription. (A) Amino acid sequence alignment of the conserved HDA-2 from Neurospora crassa, Saccharomyces cerevisiae, Drosophila melanogaster, Mus musculus, and Homo sapiens. (B) Growth of the WT, *hda-2^KO^*, and HDA-2 transformants in plates under the treatment of 0, 5, or 10 mM H_2_O_2_. The relative rate of growth was determined. (C) Quantitative results for relative growth rate of the WT, *hda-2^KO^*, and HDA-2 transformants. Error bars show SD (*n *= 3). (D) Catalase activity assay. Crude extracts from the WT, *hda-2^KO^*, and HDA-2 transformants were subjected to native-PAGE, and the catalase activities were determined by the in-gel assay. (E) Western blot analysis showing the levels of CAT-3, HDA-2, and Myc-HDA-2 proteins in the WT, *hda-2^KO^*, and HDA-2 transformants. The membrane stained by Coomassie blue represents the total protein in each sample and acted as loading control for Western blotting. (F) RT-qPCR analysis showing the levels of *cat-3* mRNA in the WT, *hda-2^KO^*, and HDA-2 transformants. Error bars show SD (*n *= 3). Significance was assessed by using a two-tailed *t* test. ***, *P* < 0.001 versus WT.

When the amino acid sequence of N. crassa HDA-2 was examined in a BLAST search against protein databases, its homologs were found to be highly conserved with Homo sapiens HDAC3, Saccharomyces cerevisiae Hos2, and Drosophila melanogaster HDAC3 (Fig. 3A). To determine whether the deacetylase activity of HDA-2 protein is required for activation of *cat-3* transcription, we generated a series of HDA-2 mutants by introducing catalytic-dead point mutation (K89A, H202A/H203A, H240A/H241A, Y371F) or deletion of the zinc-binding sites (D238 V239 H240), respectively (40–42). A plate assay showed that ectopic expression of catalytic-dead Myc-HDA-2 failed to rescue the growth defect and H_2_O_2_ sensitive phenotypes of *hda-2^KO^* strains (Fig. 3B and C), indicating that the deacetylase activity of HDA-2 plays an important role in regulation of H_2_O_2_ resistance. Consistent with the phenotypes of these transformants, expression of the catalytic-dead Myc-HDA-2 significantly decreased the CAT-3 activity, CAT-3 protein, and *cat-3* mRNA levels compared to those of the WT and *hda-2^KO^*, Myc-HDA-2 strains (Fig. 3D, E, and F). Taken together, these results demonstrated that the catalytic activity of HDA-2 protein is required for activation of *cat-3* expression.

### HDA-2-containing complex can bind to the *cat-3* locus and regulate H4 acetylation.

**FIG 4:**
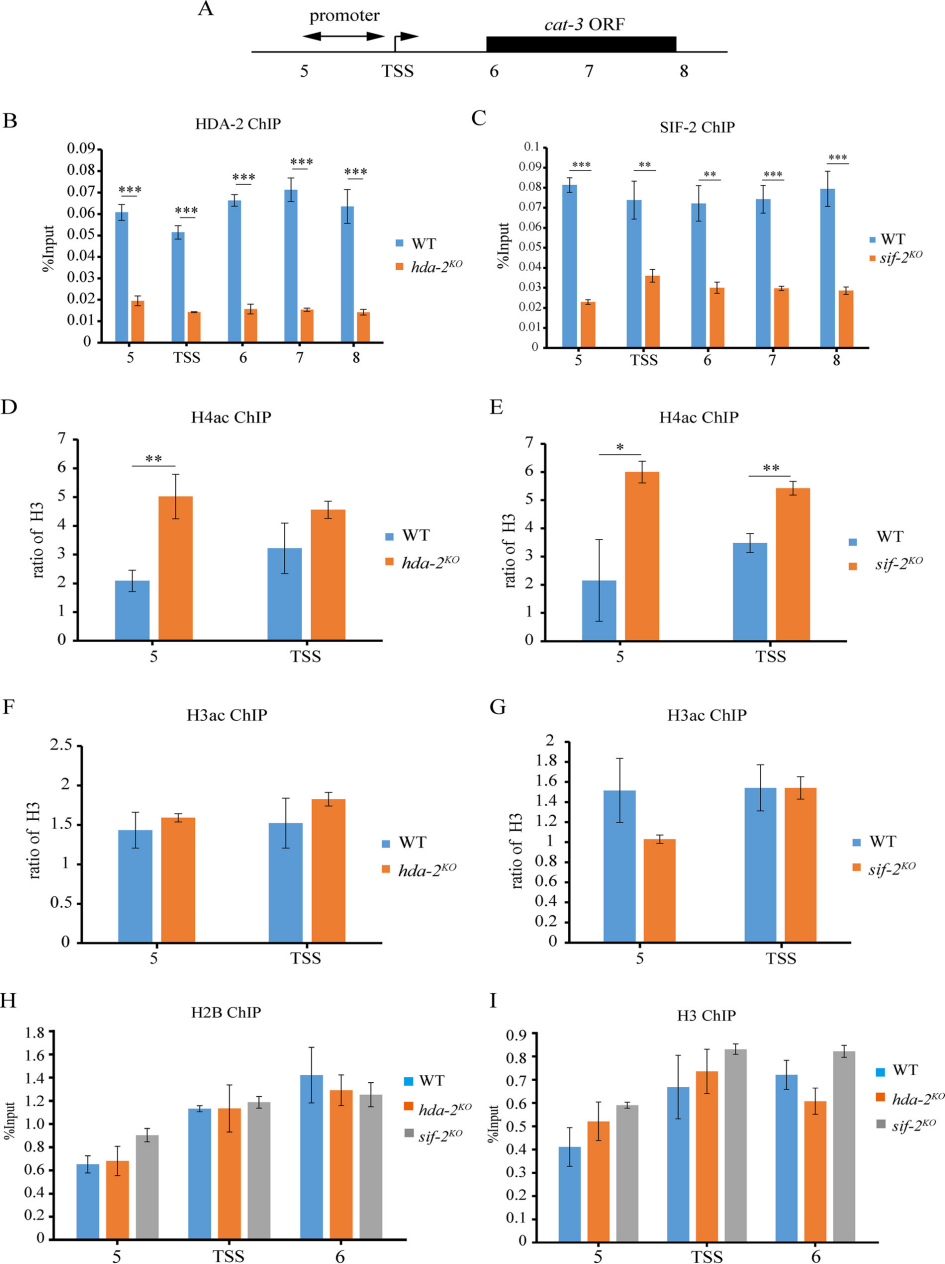
HDA-2-containing complex can bind to the *cat-3* locus and regulate H4 acetylation. (A) Schematic depiction of *cat-3* (NCU00355) on the *Neurospora* genome. (primer pairs 5 to 8) under the schematic indicate the regions tested by ChIP-qPCR. TSS, transcription start site; ORF, open reading frame. (B and C) ChIP assays showing the binding levels of HDA-2 (B) and SIF-2 (C) at the *cat-3* locus in the WT, *hda-2^KO^* (B), and *sif-2^KO^* (C) strains. (D and E) ChIP assays showing the acetylation of H4 at the *cat-3* locus in the WT, *hda-2^KO^* (D), and *sif-2^KO^* (E) strains. (F and G) ChIP assays showing the acetylation of H3 at the *cat-3* locus in the WT, *hda-2^KO^* (F), and *sif-2^KO^* (G) strains. (H and I) ChIP assays showing the occupancy levels of H2B (H) and H3 (I) at the *cat-3* locus in the WT, *hda-2^KO^*, and *sif-2^KO^* strains. Error bars show SD (*n *= 3). Significance was assessed by using a two-tailed *t* test. *, *P < *0.05; **, *P < *0.01; ***, *P* < 0.001.

To test whether HDA-2-containing complex binds to the *cat-3* locus, we carried out ChIP assays with HDA-2 or SIF-2 antibody. ChIP data revealed that HDA-2 and SIF-2 are highly enriched at the *cat-3* locus in the WT strain compared to those in the *hda-2^KO^* or *sif-2^KO^* strains (Fig. 4A to C), confirming that HDA-2-containing complex could bind to the *cat-3* locus. To test whether the binding of HDA-2 influences the histone acetylation state, we measured the levels of H3 and H4 acetylation at the *cat-3* locus in the WT, *hda-2^KO^*, and *sif-2^KO^* strains. ChIP data showed that the H4 acetylation levels were increased at the *cat-3* 5′ end of the regulation region in these mutants compared to those of WT strain (Fig. 4D and E), while the H3 acetylation levels had no significant change (Fig. 4F and G). We also measured the levels of H2B and H3 at the *cat-3* locus in the WT, *hda-2^KO^*, and *sif-2^KO^* strains. However, ChIP results showed that the occupancies of histone H2B and H3 at the *cat-3* locus were identical in WT, *hda-2^KO^*, and *sif2^KO^* strains, indicating that the nucleosome density is not affected by the binding of HDA-2 and SIF-2 at the *cat-3* locus (Fig. 4H and I). Taken together, these results suggest that HDA-2-containing complex is involved in regulation of H4 acetylation at the *cat-3* locus but has no effect on the nucleosome density.

### HDA-2-containing complex is required for PIC assembly at the *cat-3* locus.

**FIG 5:**
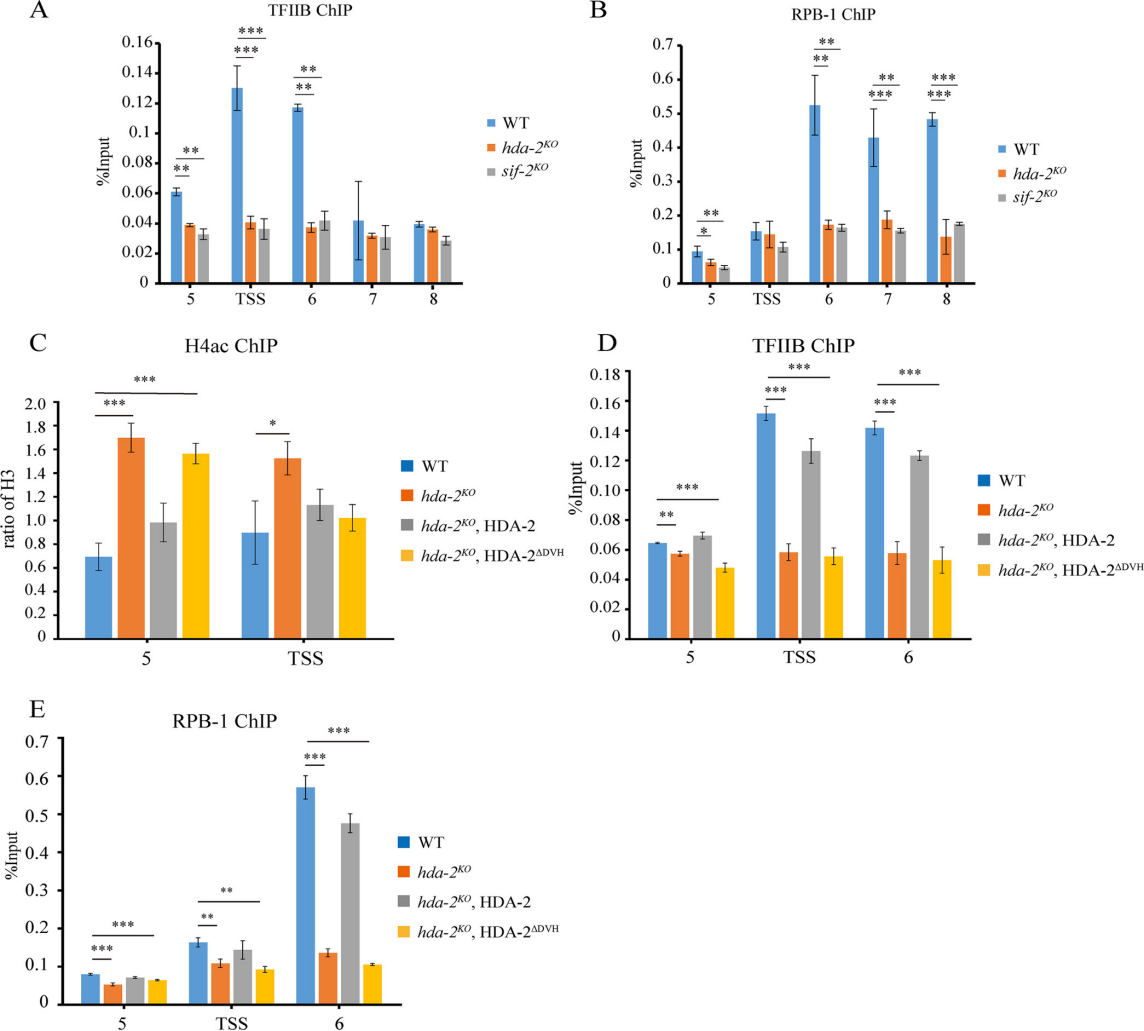
HDA-2-containing complex is required for PIC assembly at the *cat-3* locus. (A and B) ChIP assays showing the binding of TFIIB (A) and RPB-1 (B) at the *cat-3* locus in the WT, *hda-2^KO^*, and *sif-2^KO^* strains. (C) ChIP assay showing the acetylation of H4 at the *cat-3* locus in the WT, *hda-2^KO^*, and HDA-2 transformants. (D and E) ChIP assays showing the binding of TFIIB (D) and RPB-1 (E) at the *cat-3* locus in the WT, *hda-2^KO^*, and HDA-2 transformants. Error bars show SD (*n *= 3). Significance was assessed by using a two-tailed *t* test. *, *P < *0.05; **, *P < *0.01; ***, *P* < 0.001.

In S. cerevisiae, Rpd3 and Hos2 are required for RNA polymerase II (RNAP II) recruitment to the *RNR3* gene for its transcription (43). To test whether the HDA-2-containing complex is required for the PIC assembly at the *cat-3* promoter, we performed a ChIP assay using (TFIIB)-specific antibody in WT, *hda-2^KO^*, and *sif-2^KO^* strains. As shown in Fig. 5A, the enrichment of TFIIB at the *cat-3* promoter, transcription start site (TSS), and 5′ end of the open reading frame (ORF) region was dramatically decreased in *hda-2^KO^* and *sif-2^KO^* strains compared to that in the WT strain, indicating that deletion of the *hda-2* or *sif-2* gene affects binding of TFIIB at the *cat-3* locus. We further checked the enrichment of RNAP II at the *cat-3* locus in WT, *hda-2^KO^*, and *sif-2^KO^* strains. ChIP assays using RPB-1-specific antibody revealed that RNAP II enrichment was also dramatically decreased at the *cat-3* promoter and ORF regions in *hda-2^KO^* and *sif-2^KO^* strains compared to that in the WT strain (Fig. 5B), indicating that the HDA-2-containing complex facilitates the assembly of RNAP II at the *cat-3* promoter/TSS region.

A previous study showed that Rpd3 and Hos2 regulate the activation of *RNR3* by deacetylating nucleosomes at the promoter in S. cerevisiae (43). To further confirm the catalytic activity of HDA-2 in regulation of PIC assembly at the *cat-3* promoter and TSS regions, we examined the acetylation status of H4 at the *cat-3* locus in WT, *hda-2^KO^*, *hda-2^KO^*, Myc-HDA-2 or *hda-2^KO^*, Myc-HDA-2^ΔDVH^ strains. Consistent with the levels of *cat-3* expression, ChIP assays revealed that ectopic expression of Myc-HDA-2 but not Myc-HDA-2^ΔDVH^ not only recovered the low levels of H4 acetylation at the *cat-3* promoter seen in *hda-2^KO^* strain (Fig. 5C), but also significantly rescued the TFIIB and RNAP II enrichment at the *cat-3* gene region in the *hda-2^KO^* strain (Fig. 5D and E). Taken together, these data suggested that the catalytic activity of the HDA-2 protein is required for its role in activation of *cat-3* expression by regulating H4 acetylation and assembly of PIC at the *cat-3* locus.

### HDA-2-containing complex activates transcription of the *cat-3* gene by antagonizing inhibition of H2A.Z at the *cat-3* locus through H4 acetylation.

**FIG 6:**
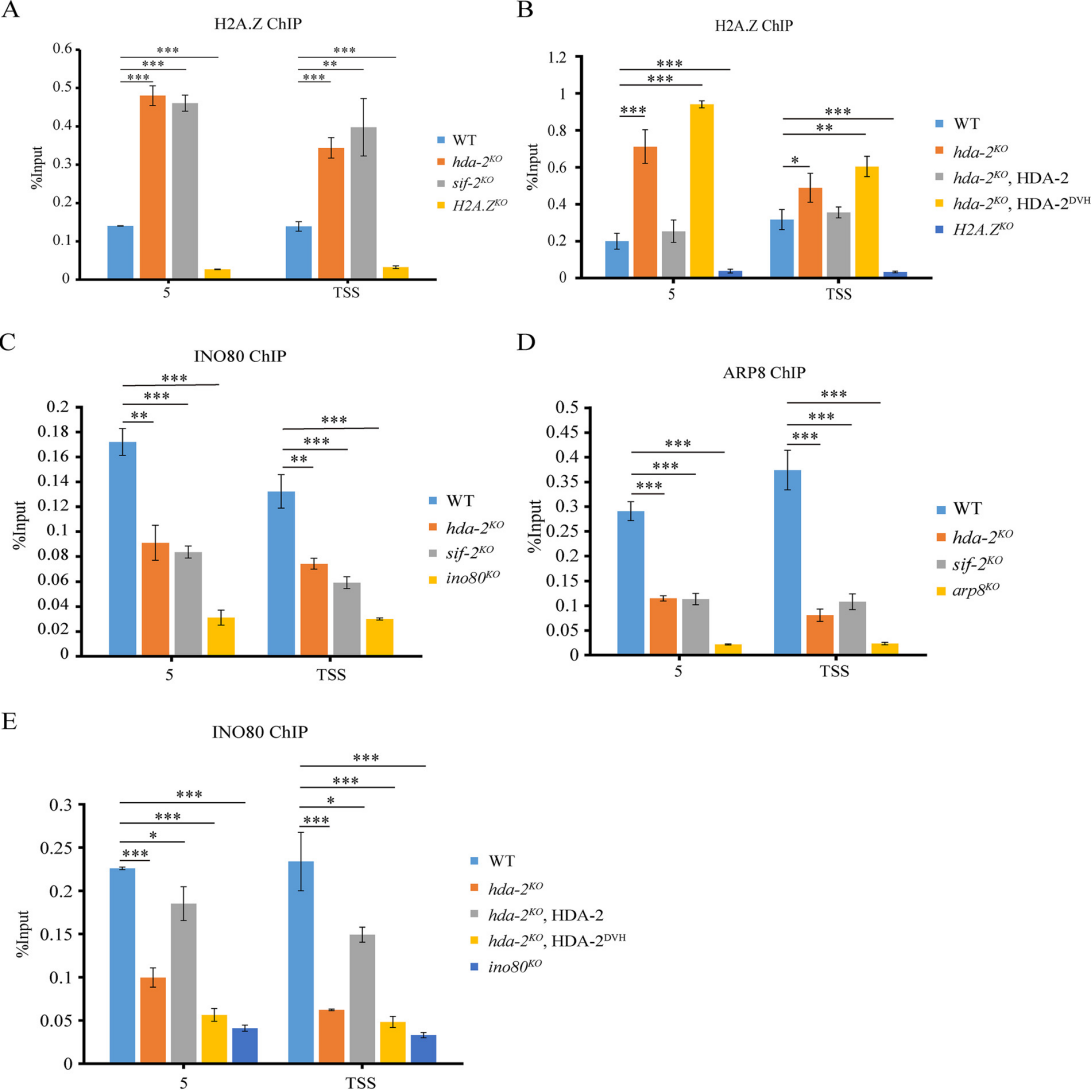
HDA-2-containing complex activates transcription of the *cat-3* gene by antagonizing the inhibition of H2A.Z at the *cat-3* locus through H4 acetylation. (A) ChIP assay showing the occupancy levels of H2A.Z at the *cat-3* locus in the WT, *hda-2^KO^*, and *sif-2^KO^* strains. The *H2A.Z^KO^* strain was used as a negative control in the ChIP assay. (B) ChIP assay showing the occupancy levels of H2A.Z at the *cat-3* locus in the WT, *hda-2^KO^*, and HDA-2 transformants. The *H2A.Z^KO^* strain was used as a negative control in the ChIP assay. (C and D) ChIP assays showing the binding of INO80 (C) and ARP8 (D) at the *cat-3* locus in the WT, *hda-2^KO^*, and *sif-2^KO^* strains. The *ino80^KO^* (C) and *arp8^KO^* (D) strains were used as negative controls in the ChIP assays. (E) ChIP assay showing the binding of INO80 at the *cat-3* locus in the WT, *hda-2^KO^*, and HDA-2 transformants. The *ino80^KO^* strain was used as a negative control in the ChIP assay. Error bars show SD (*n *= 3). Significance was assessed by using a two-tailed *t* test. *, *P < *0.05; **, *P < *0.01; ***, *P* < 0.001.

In S. cerevisiae, histone H3 and H4 tail acetylation is required for efficient recruitment of H2A.Z (36). We previously found that deposition of H2A.Z at the *cat-3* gene promoter/TSS region negatively regulated transcription of *cat-3* (22). To test whether the H4 hyperacetylation at the *cat-3* promoter/TSS region promotes H2A.Z deposition, we examined the occupancies of H2A.Z at the *cat-3* promoter/TSS region in WT, *hda-2^KO^*, and *sif-2^KO^* strains. ChIP assays using H2A.Z-specific antibody showed that H2A.Z occupancies were dramatically increased at the *cat-3* promoter/TSS region in *hda-2^KO^* and *sif-2^KO^* strains compared to that of WT strain (Fig. 6A). Moreover, ectopic expression of WT Myc-HDA-2 but not catalytic-dead Myc-HDA-2^ΔDVH^ protein rescued the increased deposition of H2A.Z at the *cat-3* promoter/TSS region in *hda-2^KO^* strain (Fig. 6B). These results indicated that HDA-2-containing complex antagonizes H2A.Z excessive deposition to activate transcription of the *cat-3* gene through H4 acetylation.

Our previous study showed that the chromatin remodeling complex INO80C positively regulates *cat-3* expression through removing H2A.Z around the *cat-3* locus (23). To test whether the recruitment of INO80C to the *cat-3* locus was affected by HDA-2-containing complex, we examined the enrichment of INO80 and ARP8, the catalytic and structural subunit of INO80C, at the *cat-3* locus in WT, *hda-2^KO^*, and *sif-2^KO^* strains. ChIP data showed that the enrichment of INO80 and ARP8 at the *cat-3* promoter/TSS region were dramatically decreased in *hda-2^KO^* and *sif-2^KO^* strains compared to those of WT strain (Fig. 6C and D). Ectopic expression of Myc-HDA-2 but not catalytic-dead Myc-HDA-2^ΔDVH^ protein significantly increased the INO80 enrichment around the *cat-3* promoter region in the *hda-2^KO^* strain (Fig. 6E). Taken together, these data demonstrated that the catalytic activity of HDA-2 protein is required for its role in activation of *cat-3* expression by regulating the INO80C-mediated removal of H2A.Z around the *cat-3* promoter/TSS region.

## DISCUSSION

Our recent studies have shown that H_2_O_2_ treatment can induce *cat-3* gene expression and increase H3 acetylation at the *cat-3* locus (20). Biochemical analysis showed that the transcription factor CPC1/GCN4 and histone acetyltransferase GCN5 are required for maintenance of *cat-3* expression and response to H_2_O_2_-induced oxidative stress in N. crassa (21). It is necessary to further identify the histone-modifying enzymes involved in regulation of *cat-3* gene expression. *Neurospora* has 11 predicted proteins related to known or putative histone deacetylases (HDACs) (13). In this study, we generated most of the deletion mutants of these HDAC genes, but not the homokaryotic deletion strains of the *nst-3* (NCU03059) or *nst-6* (NCU05973) gene. According to the catalase activity in-gel assay and Western blot analysis, we found that only HDA-2 is involved in the positive regulation of *cat-3* expression compared with the other eight histone deacetylases in N. crassa (see Fig. S4A and B in the supplemental material). In its catalytically dead mutants, the *cat-3* expression level is significantly decreased, like that in *hda-2^KO^* strains, indicating that expression of *cat-3* is dependent on the catalytic activity of HDA-2. HDA-2 is a deacetylase of the class I type histone deacetylases and is the homolog of S. cerevisiae Hos2. Hos2 and Set3, which is the homologous protein of N. crassa SET-4, are central components of the Set3 complex (24). However, deletion of the *hda-2*, *sif-2*, or *snt-1* subunit but not *set-4* or *nst-1* in the N. crassa genome leads to downregulation of *cat-3* expression. Immunoprecipitation data confirmed the interaction among N. crassa HDA-2, SIF-2, and SNT-1 proteins. Taken together, our results suggest that HDA-2, SIF-2, and SNT-1 proteins may form a subcomplex for *cat-3* activation.

10.1128/mbio.01351-22.4 FIG S4 **Only HDA-2 is involved in positive regulation of *cat-3* expression compared with the other eight histone deacetylases in** **N. crassa.** (A) Catalase activity in-gel assay. Crude extracts from the WT, *hda-1^KO^*, *hda-2^KO^*, *hda-3^KO^*, *hda-4^KO^*, *nst-1^KO^*, *nst-2^KO^*, *nst-4^KO^*, *nst-5^KO^*, and *nst-7^KO^* strains were subjected to native-PAGE, and the catalase activity was determined with the in-gel assay. (B) Western blot analysis showing the levels of CAT-3 protein in the WT, *hda-1^KO^*, *hda-2^KO^*, *hda-3^KO^*, *hda-4^KO^*, *nst-1^KO^*, *nst-2^KO^*, *nst-4^KO^*, *nst-5^KO^*, and *nst-7^KO^* strains. The membrane stained with Coomassie blue represents the total protein in each sample and acted as a loading control for Western blotting. Download FIG S4, TIF file, 1.0 MB. Copyright © 2022 He et al. 2022 He et al. https://creativecommons.org/licenses/by/4.0/ This content is distributed under the terms of the Creative Commons Attribution 4.0 International license.

The role of HDA-2-containing complexes in yeast and mammals in gene expression has been reported in previous studies. As a histone deacetylase, we found that HDA-2-containing complex positively regulates *cat-3* transcription in N. crassa. In agreement with this result, HDA-2 was identified as a positive regulator for *cat-3* transcription in Trichoderma atroviride (39). In yeast, Hos2 is important for activation of *GAL1* and *INO1* genes through binding to the coding regions of genes (25). In addition, Hos2 and an associated factor, Set3, are necessary for efficient gene transcription (25). In contrast to other class I histone deacetylases, which are frequently found as corepressors, Hos2 is directly required for gene activation. To identify the underlying molecular mechanism of HDA-2 for *cat-3* transcription, we performed a series of experiments and found that histone H4 acetylation but not H3 acetylation at the *cat-3* locus in *hda-2^KO^* and *sif-2^KO^* strains was increased. A previous study showed that H4 acetylation could affect the assembly of PIC (44). To be specific, a previous work reported that Cmr1, a largely uncharacterized nuclear protein in S. cerevisiae, is recruited to regulate RNAP II occupancy in transcribed coding regions, which is stimulated by the histone deacetylases Rpd3 and Hos2 (45). In this study, we found that deletion of HDA-2 or SIF-2 lead to a dramatic decrease of TFIIB and RPB-1 levels at the *cat-3* promoter and TSS regions (Fig. 5A and B). These results strongly suggest that the HDA-2-mediated deacetylation of H4 is necessary for efficient recruitment of transcriptional machinery for activation of the *cat-3* gene.

We previously showed that H2A.Z is immediately evicted from the chromatin at the *cat-3* locus in response to oxidative stress with a corresponding accumulation of CPC1 at the *cat-3* locus, and we suggested that H2A.Z antagonizes CPC1 binding to restrict *cat-3* expression in a normal setting, whereas under oxidative stress H2A.Z is removed from chromatin, leading to a rapid and full activation of *cat-3* transcription (22). In addition, our recent study found that the negative cofactor 2 (NC2) complex activates *cat-3* expression by recruiting INO80 complex to remove H2A.Z from special H2A.Z-containing nucleosomes at the promoter and TSS of the *cat-3* gene (23). Furthermore, NC2 is involved in removal of H2A.Z at *hsp70/dnak* and *hsp90a* genes (23). These results suggest that H2A.Z deposition may serve as a regulatory target for external stimuli and that the structure of H2A.Z-containing nucleosomes around these inducible genes is less stable than those of H2A-containing nucleosomes. In budding yeast, efficient deposition of H2A.Z is further promoted by histone H3 and H4 tail acetylation and the bromodomain protein Bdf1, a component of the SWR1 remodeling complex that deposits H2A.Z (36). It has been found in previous studies that there is an interplay between the SWR1 complex and INO80 complex. For example, AtARP6, a specific subunit of SWR1-C that mediates the H2A.Z exchange in *Arabidopsis*, was found to have an inhibitory role in the local chromatin enrichment of AtINO80 (46). Moreover, both *htz1Δ set3Δ* and *swr1Δ set3Δ* exhibit a severe slow growth phenotype in S. cerevisiae, but the *htz1Δ swr1Δ set3Δ* triple mutant grows relatively well, indicating Set3/Hos2 histone deacetylase has previously unrecognized functions in dynamic deposition and remodeling of nucleosomes containing H2A.Z (38). Our ChIP assays showed that the abundance of H2A.Z at the *cat-3* locus was dramatically increased in the absence of HDA-2 and SIF-2. In contrast, loss of HDA-2 and SIF-2 resulted in a decreased recruitment of INO80 and ARP8 subunits of the INO80 complex at the *cat-3* locus compared to that in the WT strain. These results indicated that HDA-2-containing complex positively regulates transcription of the *cat-3* gene by antagonizing inhibition of H2A.Z at the *cat-3* locus through H4 acetylation.

According to our previous study, the H2A.Z-containing nucleosomes should be removed upon gene induction to provide access for the transcriptional machinery (22). In yeast, a genome-wide study showed that blocking PIC assembly resulted in promoter-specific H2A.Z accumulation, while H2A.Z eviction was unaffected upon depletion of INO80, indicating that the PIC is required to evict H2A.Z (47). In this study, we found that HDA-2-containing complex is required not only for PIC assembly but also for antagonizing the inhibition of H2A.Z at the *cat-3* locus through H4 acetylation. Therefore, these results cannot completely exclude an interplay between PIC defective assembly and H2A.Z excessive deposition at the *cat-3* locus in *hda-2^KO^* and *sif-2^KO^* strains.

## MATERIALS AND METHODS

### Strains and culture conditions.

The 87-3 (*bd*, *a*) strain was used as the wild-type strain in this study (48). The *ku70 ^RIP^* (*bd*, *a*) strain, generated previously (49), was used as the host strain for creating the *hda-2* (NCU02795), *sif-2* (NCU06838), *snt-1* (NCU10346), *nst-1* (NCU04737), and *set-4* (NCU4389) knockout mutants by deleting the entire ORF through homologous recombination using a protocol described previously (50). The plasmid containing the *cfp* promoter driven the HDA-2 ORF and its 3′-untranslated region (pcfp-5Myc-6×His-HDA-2) was used as the template for mutagenesis, and five mutations of HDA-2 (HDA-2^ΔDVH^, HDA-2^K89A^, HDA-2^H240A/H241A^, HDA-2^H202A/H203A^, and HDA-2^Y371F^) were generated. Afterwards, plasmids pcfp-5Myc-6×His-HDA-2, pcfp-5Myc-6×His-HDA-2^ΔDVH^, pcfp-5Myc-6×His-HDA-2^K89A^, pcfp-5Myc-6×His-HDA-2^H240A/H241A^, pcfp-5Myc-6×His-HDA-2^H202A/H203A^, and pcfp-5Myc-6×His-HDA-2^Y371F^ were transformed into *hda-2^KO^* (*bd*, *his-3*-deficient) strains to obtain the transformants *hda-2^KO^*, Myc-HDA-2, *hda-2^KO^*, Myc-HDA-2^ΔDVH^, *hda-2^KO^*, Myc-HDA-2^K89A^, *hda-2^KO^*, Myc-HDA-2^H240A/H241A^, *hda-2^KO^*, Myc-HDA-2^H202A/H203A^, and *hda-2^KO^*, Myc-HDA-2^Y371F^. Applying the same method, a *sif-2^KO^*, Myc-SIF-2 strain and *snt-1^KO^*, Myc-SNT-1 strain were created. All strains used in this study possess the same *bd* background.

The medium for plate assays contained 1× Vogel’s salts, 3% sucrose, and 1.5% (wt/vol) agar with or without H_2_O_2_. Liquid cultures were grown at 25°C with shaking in minimal medium (1× Vogel’s and 2% glucose) for 18 h in constant light (LL).

### Plate assay.

Age-appropriate conidia were inoculated in petri dishes with 50 mL liquid medium containing Vogel’s minimal medium (VM) and 2% glucose under static culture condition at 25°C in constant light (LL) until the exponential growth phase of mycelium. The disks of mycelium mat were cut with a cork borer for quantification. For each strain, an individual mycelium disk was transferred into the centers of VM plates containing 3% sucrose and 1.5% (wt/vol) agar and cultured at 25°C in constant light (LL). The response to oxidative stress was determined by analyzing disk diameters of strains on VM plates containing 3% sucrose and 1.5% (wt/vol) agar with or without H_2_O_2_ at the indicated concentrations. In order to exclude the effect of the growth rate of different strains on the H_2_O_2_ sensitivity, the calculation method used previously, which included the extent of relative growth rate to represent the extent of H_2_O_2_ sensitivity, was also used in this study (20–22). In addition, in order to visually analyze the growth phenotype, all plates were photographed until the disk diameters of the wild-type strain in medium without H_2_O_2_ exactly extended to the edge of the plate. Then, we directly analyzed the H_2_O_2_ sensitivity through visual observation of the disk diameters of strains in medium with oxidative stress.

### In-gel assay for catalase.

Cell extracts of mycelium disks cultured for 18 h in liquid medium were used for the zymogram. Ground tissues were mixed with ice-cold extraction buffer containing 50 mM HEPES (pH 7.4), 137 mM NaCl, 10% glycerol, and protease inhibitors pepstatin A (1 μg/mL), leupeptin (1 μg/mL), and phenylmethylsulfonyl fluoride (PMSF; 1 mM), and centrifuged at 10,000 × *g* for 10 min at 4°C. The protein concentration was measured by Bio-Rad protein assay dye at 595 nm. For the in-gel assay, catalase activity was determined as described previously (51). Equal amounts of total protein (40 μg) were loaded into a 7.5% native polyacrylamide slab gel. After electrophoresis, the gel was immersed in 10 mM H_2_O_2_ with shaking for 10 min and then in a 1:1 mixture of freshly prepared 1% potassium hexacyanoferrate (III) and 1% iron (III) chloride hexahydrate. Catalase activity was visualized as a band where H_2_O_2_ was decomposed by catalase.

### Protein analysis.

Protein extraction, quantification, and Western blot analysis were performed as described previously (52). Equal amounts of total protein (40 μg) were loaded into each lane. After electrophoresis, proteins were transferred onto a polyvinylidine difluoride (PVDF) membrane. Western blot analysis was performed using antibodies against the proteins of interest.

### RNA analysis.

For quantitative real-time reverse transcriptase quantitative PCR (qPCR), total RNA was isolated with TRIzol reagent and treated with DNase I to remove genomic DNA, according to the manufacturer’s protocol. Each RNA sample (total RNA, 5 μg) was subjected to reverse transcription with Moloney murine leukemia virus reverse transcriptase (Promega) and then amplified by real-time PCR (ABI 7500). The primers used for qPCR are shown in Table S1 in the supplemental material. The relative values of gene expression were calculated using the threshold cycle (2^−ΔΔ^*^CT^*) method (53) by comparing the cycle number for each sample with that for the untreated control. The results were normalized to expression levels of the β-tubulin gene.

10.1128/mbio.01351-22.5 TABLE S1 Primers for RT-qPCR assays. Download Table S1, DOCX file, 0.01 MB. Copyright © 2022 He et al. 2022 He et al. https://creativecommons.org/licenses/by/4.0/ This content is distributed under the terms of the Creative Commons Attribution 4.0 International license.

### Generation of antiserum against HDA-2 and SIF-2.

Glutathione *S*-transferase (GST)–HDA-2 (amino acids T384 to R487) and GST-SIF-2 (amino acids E109 to N347) fusion proteins were expressed in BL21 cells, and soluble recombinant proteins were purified and used as the antigens to generate rabbit polyclonal antiserum, as described previously (54, 55).

### ChIP analysis.

Chromatin immunoprecipitation (ChIP) assays were performed as described previously (56). Briefly, N. crassa tissues were fixed with 1% formaldehyde for 15 min at 25°C with shaking. Glycine was added at a final concentration of 125 mM, and samples were incubated for another 5 min. The cross-linked tissues were ground and resuspended at 0.5 g in 6 mL lysis buffer containing protease inhibitors (1 mM PMSF, 1 μg/mL leupeptin, and 1 μg/mL pepstatin A). Chromatin was sheared by sonication to approximately 500- to 1,000-bp fragments. A 1-mL aliquot of protein solution (2 mg/mL) was used for each immunoprecipitation reaction, and 10 μL was kept as the input DNA. The ChIP was carried out with 3 μL of anti-H4ac antibody (06-866; Millipore), 3 μL of anti-H3ac antibody (06-599; Millipore), 3 μL of anti-H3 antibody (2650; CST), 2 μL of anti-H2B antibody (1790; abcam), 10 μL of anti-HDA-2 antibody, 10 μL of anti-SIF-2 antibody, 10 μL of anti-TFIIB antibody, 10 μL of anti-RPB-1 antibody, 10 μL of anti-H2A.Z antibody, 10 μL of anti-INO80 antibody, 10 μL of anti-ARP8 antibody. Immunoprecipitated DNA was quantified by using real-time PCR with primer pairs. The primer pairs used are listed in Table S2 in the supplemental material. ChIP-quantitative PCR data were normalized by the input DNA and are presented as a percentage of input DNA. Each experiment was independently performed at least three times.

10.1128/mbio.01351-22.6 TABLE S2 Primers for ChIP-qPCR assays. Download Table S2, DOCX file, 0.01 MB. Copyright © 2022 He et al. 2022 He et al. https://creativecommons.org/licenses/by/4.0/ This content is distributed under the terms of the Creative Commons Attribution 4.0 International license.

### Coimmunoprecipitation.

Cell extracts from the adhered mycelium mat incubated for 18 h were used for performing coimmunoprecipitation (co-IP) analyses. Protein extraction, quantification, and coimmunoprecipitation assays were performed as described previously (55). Briefly, 4-mg/mL protein extracts in extraction buffer were incubated with 5 μL of monoclonal antibody to c-Myc (HT101-02; TransGen Biotech), 10 μL of antibody to HDA-2, and/or 10 μL of antibody to SIF-2 for 4 h at 4°C with rotation. Then, the 40 μL of precleaned protein G-Sepharose (17-0885-02; GE Healthcare) was added and incubated for 1 h at 4°C with rotation. The beads were washed three times with ice-cold extraction buffer, mixed with protein loading buffer, and boiled for 10 min, and the immunoprecipitated proteins were analyzed by Western blotting.
