# Supplementary figures and images for: HDA-2-Containing Complex Is Required for Activation of Catalase-3 Expression in Neurospora crassa
Source: mBio. 2022 Jun 14;13(4):e01351-22. doi: 10.1128/mbio.01351-22 (PMC9426557; doi:10.1128/mbio.01351-22)

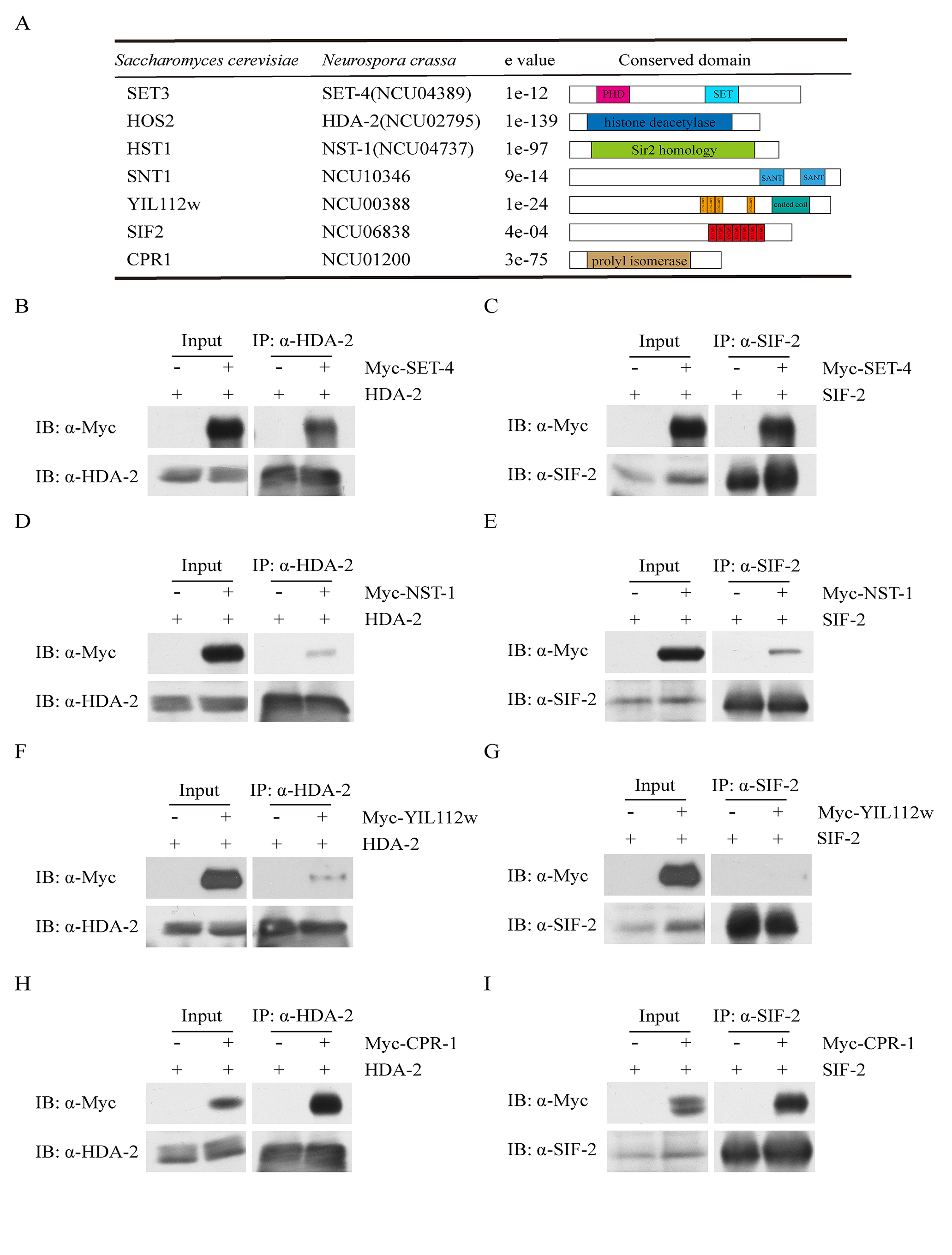

Supplement: FIG S1 [file mbio.01351-22-s0001.tif]

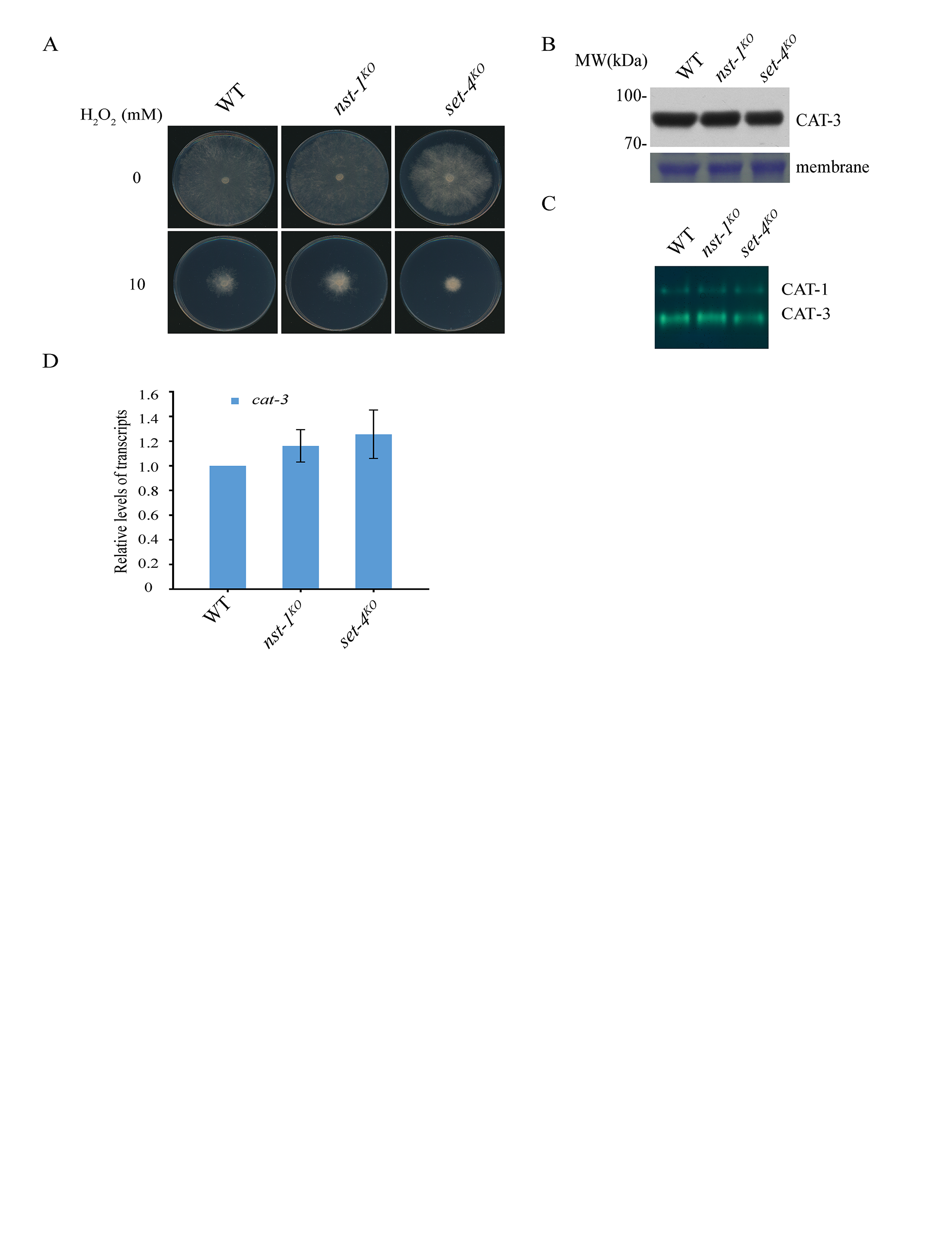

Supplement: FIG S2 [file mbio.01351-22-s0002.tif]

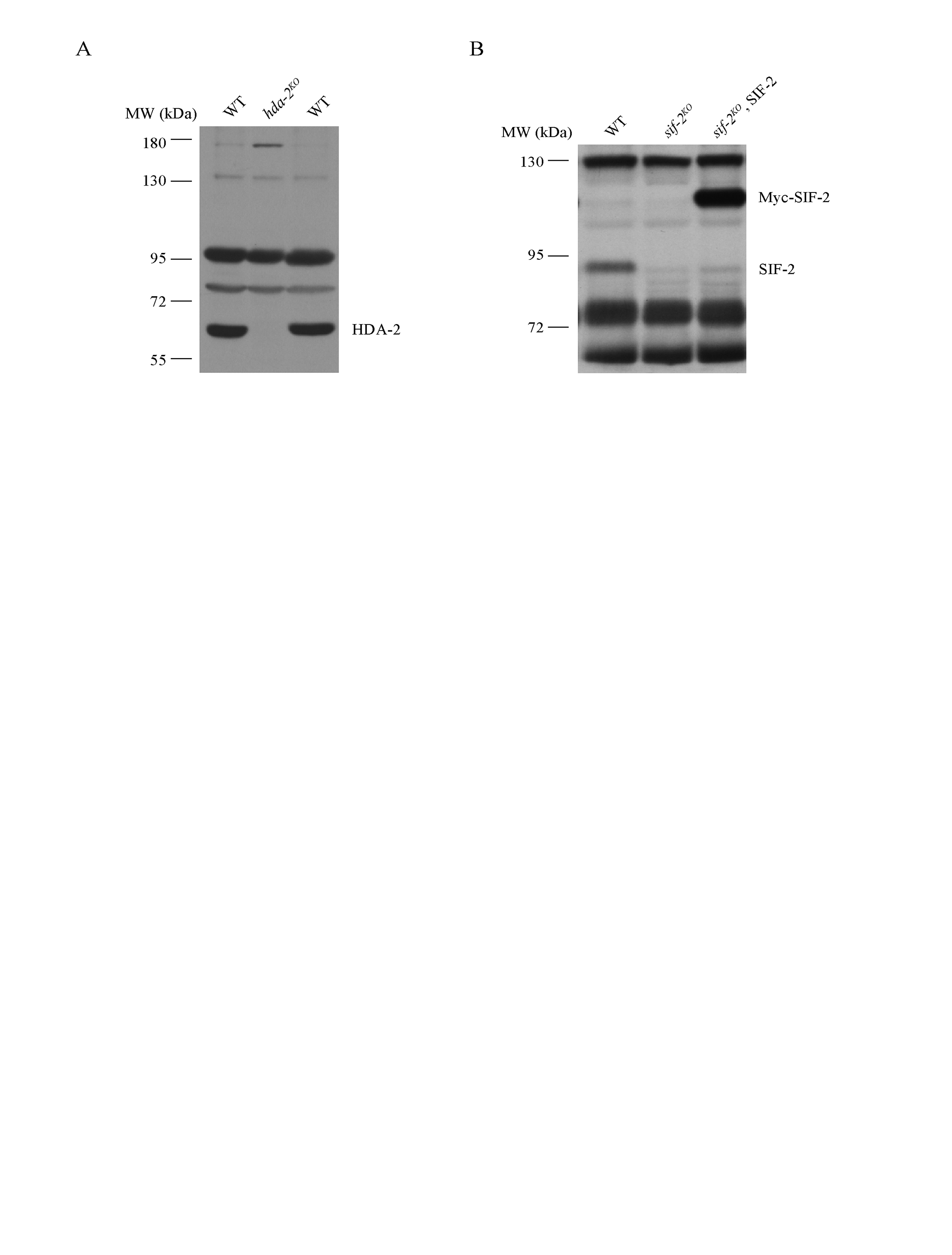

Supplement: FIG S3 [file mbio.01351-22-s0003.tif]

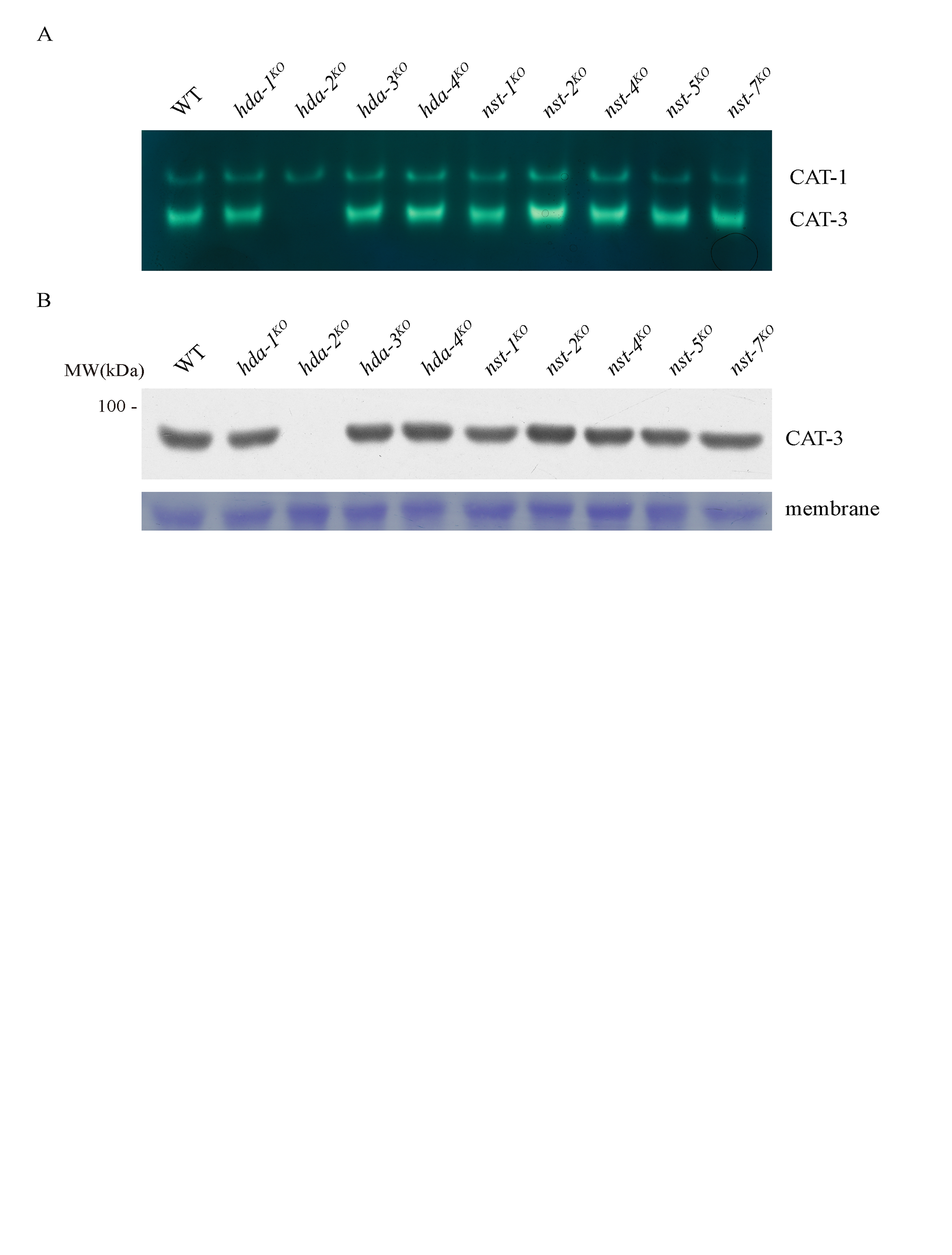

Supplement: FIG S4 [file mbio.01351-22-s0004.tif]
