# Supplementary material for: HDA-2-Containing Complex Is Required for Activation of Catalase-3 Expression in Neurospora crassa
Source: mBio. 2022 Jun 14;13(4):e01351-22. doi: 10.1128/mbio.01351-22 (PMC9426557; doi:10.1128/mbio.01351-22)
Supplement: TABLE S1 [file mbio.01351-22-s0005.docx]

Supplementary Table S1

| Primes for RT-qPCR | Sequence (5ʹ to 3ʹ) |
| --- | --- |
| *cat-3. F* | GCCGTCCTAGCCAGATTCTTAC |
| *cat-3. R* | ACTCCTCATCATCGCCATCAAC |
| *β-tubulin. F* | GCGTATCGGCGAGCAGTT |
| *β-tubulin. R* | CCTCACCAGTGTACCAATGCA |
