# Supplementary material for: HDA-2-Containing Complex Is Required for Activation of Catalase-3 Expression in Neurospora crassa
Source: mBio. 2022 Jun 14;13(4):e01351-22. doi: 10.1128/mbio.01351-22 (PMC9426557; doi:10.1128/mbio.01351-22)
Supplement: TABLE S2 [file mbio.01351-22-s0006.docx]

Supplementary Table S2

| Primes for ChIP-qPCR | Sequence (5ʹ to 3ʹ) |
| --- | --- |
| *cat-3* 5F | TCTTGTAGATTTGGTGTCAGAGG |
| *cat-3* 5R | GGTGCCTTGGTTTTGGTTGC |
| *cat-3* 6F | TTGTCACACATTCTCCCTGTC |
| *cat-3* 6R | CCAATAAGGCCGCTCAACG |
| *cat-3* 7F | TCAAGTCCTCGCCGGCAAG |
| *cat-3* 7R | GGATCAAGAAGGTCGAAGCC |
| *cat-3* 8F | CTTACTGATGGTTACCGCTGG |
| *cat-3* 8R | CCTTGATAACCTCGTCCTGAG |
| *cat-3* TSS | GAACCCGTTCTCCAAGTACC |
| *cat-3* TSS | CTTTGCCAGGATCAACACAAC |
